# Can Stephen Curry really know?—Conscious access to outcome prediction of motor actions

**DOI:** 10.1371/journal.pone.0250047

**Published:** 2022-01-18

**Authors:** Lisa Katharina Maurer, Heiko Maurer, Mathias Hegele, Hermann Müller

**Affiliations:** 1 Department of Psychology and Sport Science, Neuromotor Behavior Lab, Justus Liebig University, Giessen, Germany; 2 Center for Mind, Brain and Behavior (CMBB), Universities of Marburg and Giessen, Giessen, Germany; Texas A&M University, UNITED STATES

## Abstract

The NBA player Stephen Curry has a habit of turning away from the basket right after taking three-point shots even before the ball reaches the basket, suggesting that he can reliably predict whether the just released shot will hit or not. In order to use this “knowledge” to deliberately decide which action to take next, Stephen Curry needs conscious access to the results of internal processes of outcome prediction and valuation. In general, computational simulations and empirical data suggest that the quality of such internal predictions is related to motor skill level. Whether the results of internal predictions can reliably be consciously accessed, however, is less clear. In the current study, 30 participants each practiced a virtual goal-oriented throwing task for 1000 trials. Every second trial, they were required to verbally predict the success of the current throw. Results showed that on average, verbal prediction accuracy was above an individually computed chance level, taking into account individual success rates and response strategies. Furthermore, prediction accuracy was related to task skill level. Participants with better performances predicted the success of their throws more accurately than participants with poorer performances. For the poorer performing individuals, movement execution was negatively affected when the verbalized predictions were required. They also showed no noticeable modulation of speech characteristics (response latency) for correct and incorrect predictions as observed in the high performers.

## 1 Introduction

There is anecdotal evidence that motor experts are aware of their own movement errors and can indicate them (by verbal report or gestures) even before they perceive any external feedback. These alleged predictive abilities of, for instance, NBA players like Stephen Curry can be observed in both professional and amateur game videos. These clips show that shortly after the ball leaves the players’ hand, the players already cheer in cases of successful throws or express their disappointment in cases of throws that are going to miss. But is it really possible to predict the outcome of a movement as highly complex as basketball shooting with sufficient precision based solely on information about movement preparation and execution? In order to answer this, it is necessary to (a) briefly explain how predictions about movement outcomes (successful movements or unsuccessful erroneous movements) can come about, and (b) how it is possible to verbalize or otherwise indicate these predictions.

Theories of internal models claim that forward models in the motor system are a set of neural processes that integrate information from the current state of the system and its environment including motor (efferent) commands and sensory signals from movement execution to predict sensory consequences of that movement [1–4]. These sensory consequences of motor commands can refer to parameters of movement execution or the movement outcome itself. Motor errors, within this context, are differences between *desired* and *actual* sensory outcomes of a movement. Hence, forward models are capable of predicting motor errors before they occur (or before they are observable). The advantages of the use of forward models in motor control are numerous [5]: They allow the production of fast, dexterous movements because delays in neural signal transduction can be compensated. Furthermore, sensory and motor noise increase the uncertainty in the state estimate of the system. Forward models act as filters, capable of reducing this uncertainty and attenuating unwanted information, or highlighting information critical for control. With this, it is further possible to discriminate self-motion from being moved by another agent. Lastly, forward models are considered to generate accurate and appropriate motor behavior under many different and often uncertain environmental conditions (context estimation).

Neurophysiological studies associate specific brain activity with predictive processes of error detection by event-related EEG potentials that emerge prior to the presentation of outcome feedback in simple choice reaction time tasks (e.g., [6–8]. Thus, these correlates indicate internal comparisons of *desired* with *predicted* movement outcomes on the basis of efferent information about motor commands. In cases of delayed movement outcomes (with respect to movement termination) like in basketball shots, efferent information can even be integrated with incoming sensory information from movement execution to yield a more reliable outcome estimate [2,9,10]. In accordance with this, findings of predictive error-related neural correlates in force-field reaching [11], throwing [12,13], force production [14], and ballistic pointing [15] indicate that the human motor system makes internal predictions about movement outcomes also in rather complex tasks. It is assumed that these prediction processes are tightly interwoven with ongoing motor control at different hierarchical levels, with substantial components being located in the cerebellum. This leads to the question, how these at least to some extent “deep” internal predictions become reportable to the performer? For internal processes to be reportable, they need to enter conscious attention or awareness. The term consciousness may refer to different aspects, such as “momentary subjective visual [or in general perceptual] experience as opposed to unconscious processing, reportability, or the stream of consciousness as it evolves over time, response patterns of subjects reporting on their visual [perceptual] experience, and potentially many other concepts” [16, p. 2]. In the present study, we focus on the reportability of perceptual experience, hence, we use “conscious access” referring to the ability to report contents of perceptual states.

Importantly, perception is not limited to the processing of sensory (afferent) signals, as perception can also arise from efferent information about motor commands. It is not the goal of the current study to explain how internal prediction processes become conscious perceptual experiences or how unconscious processes drive conscious experience. Rather, we aim to answer the question of whether conscious access to the described internal prediction processes is possible in complex tasks such as basketball shooting, that is, whether they can be reported or otherwise expressed as suggested by the showy behavior of top athletes. Still, theories on conscious perception posit that predictions play an important role in consciousness. For instance, according to Hohwy [17] perception may become conscious experience when there is either a strong incoming error signal that boosts attention, or when internal predictions increase the gain of expected sensory signals. The latter case implicates that a forward model produces reliable predictions. This goes along with claims of the internal model theories that forward models play an essential role in motor learning [18]. Based on computational simulations, it is suggested that learning is faster if the forward model is better able to model the dynamics of the movement and its effects [19]. In line with this, empirical data confirm that processing and valuation of motor errors based on forward model predictions is strongly related to learning and skill level. First, it has been shown that neurophysiological correlates of predictive error valuation increase with learning [20–22]. Second, participants with extended experience in a throwing task show more distinct signs of predictive error valuation on the neurophysiological level [12,13]. From this, it can be assumed that people with reliably working forward models, in case they would indeed have conscious access to the output of their forward model, should be able to verbally predict the outcome of a motor action before any external feedback about this outcome is available. Studies on metacognition and perceptual anticipation support this assumption. The following briefly describes the findings of these studies and outlines open questions and aspects to be considered.

Arbuzova and colleagues [23] examined metacognitive abilities in the discrimination of two different outcomes based on predictions in a similar version of the virtual goal-oriented throwing task used here. After each trial, participants had to decide which of two alternative task outcomes had been produced by their own movement. Results from this study do not allow conclusions about effect prediction (because discrimination accuracy was fixed in the task design), but, confidence about the discrimination ratings (metacognitive ability) was relatively high across different informational domains (visual, visuomotor, motor). This shows that aspects of one’s own movement execution are principally available for conscious use, at least with respect to the monitoring of performance. Yet, accessibility of information does not necessarily imply reportability. Reportability of sensorimotor prediction with respect to action outcomes has been investigated in athletes involved in different sports. The focus of most studies has been on anticipatory estimates of other players’ actions (instead of their own actions), based purely on visual information, for example in basketball shooting [24–27], volleyball smashes [28,29], soccer penalty kicks [30], or other game situations (for a review see [31]. Such perceptual anticipative abilities are suggested to depend on predictive motor simulations of the forward model triggered by the observation of the behavior of others [31–33]. Although, the reported predictions in the above listed studies were exclusively based on visual information, expert performers (athletes) in the corresponding sports showed better predictive abilities than expert watchers (journalists, coaches) or novices. Other studies examined predictions in both observers and performers [34], or in performers alone [35,36]. In the studies of Cañal-Bruland et al. and Maglott and colleagues, performers had to rate outcomes of basketball shots after their vision was occluded by shutter googles immediately after ball release. Results showed that, on average, performers were able to verbally predict the results of their shots above the level of chance. Both studies reported a strong judgment bias regarding shooting position and outcome (hits vs. misses), that was taken into account when calculating the base rate (chance level) of correct judgements. Yet, the base rate reflecting pure chance also depends on the actual individual hit rate, which, however, was not included in the base rate estimations provided by the authors. Furthermore, in both studies, the shutter googles were manually controlled, which presumably introduced relatively large temporal variations of the occlusions, and may have allowed participants to perceive and process post-release information about ball trajectory. Gray and colleagues [36] examined outcome prediction in virtual baseball batting, in which participants had to mark the landing position of the ball on a screen. They found that expert players showed less radial error in predicting ball location than less experiences players. Outcome predictions in this study were, however, confounded with feedback information. Participants received feedback about the final landing location of the ball and thereby the accuracy of their predictions after every trial. Thus, this study does not resolve whether the experts were better at predicting their batting outcomes because they had better predictive skills or because they were better able to “strategically” link feedback information about past predictions to actual performance.

The present study aimed to verify that individuals with high motor skills can verbally predict outcomes of their own actions based on internal modeling, without any external feedback about action outcomes being available to them. Note that the study was not designed to compare natural motor experts with extensive practice experience [37] to novice performers, but to conduct a laboratory study as a standardized approach to differentiate individuals in terms of their level of motor skill and to relate this to verbal prediction skills. In fact, motor expertise is also defined by other key elements than practice experience, such as consistently high performance and high precision with which movements are repeatedly retrieved and executed. However, these features can be viewed as improving continuously along the learning process [38,39]. For the experimental task, a virtual goal-oriented throwing task with parallels to basketball shooting was used. One significant advantage of studying throwing in this context is the natural delay between movement (throwing) termination and the availability of outcome feedback. Moreover, since the task was virtual and movement execution was captured online, the visual information available to subjects could be precisely controlled. Thus, outcome predictions could be based exclusively on information gathered during movement planning (efferent information) and during movement execution (haptic, proprioceptive, or visual information), but not on external information about movement effects (e.g., trajectory of the object to be thrown). Hence, information on the level of an individual’s accuracy in verbalizing outcome predictions (which we term *prediction accuracy* in the following) would provide novel insight into the quality of forward models and the easiness or efficiency of conscious access to forward models. *Prediction accuracy* was quantified by the rate of correct verbal predictions of throwing outcome, relative to a baseline (chance) level accounting for individual hit rates and response strategies.

To sum up, successful verbalized predictions require at least two separate functions: a predictor and conscious access to its predictions. Or, conversely, poor prediction accuracy may arise from two reasons: (i) individuals have poor prediction quality (due to poor forward models), and/or (ii) they have difficult or inefficient conscious access to their forward models. Thus, it is expected that if individuals with superior throwing performances (inferring good forward models) had easy conscious access to internal processes, they would be able to predict their throwing outcomes above the level of chance. In the present study design, the integrated effect of both factors (prediction quality and ease of access) was examined. Experimental separation to conclusively decide which of the two factors contributes to the observed differences and to what extent was not directly possible. However, we will provide post-hoc interpretations regarding the different influences of prediction quality (i) and ease of access (ii) on prediction accuracy based on the relation of skill-level and predictive abilities (e.g., [24]) and on additional variables contributing to this differentiation: throwing performance and speech characteristics of verbal responses. A possible back wash effect of conscious access to internal prediction processes results in interference with throwing performance. The preparation of verbal predictions to be performed after movement execution might disrupt the motor control process of movement execution, and hence affect throwing performance. It is postulated that conscious attention to the execution of well-practiced, movements can impair this skill [40–43]. In the present study, these costs of verbal prediction should be observable in performance differences between throws with verbal predictions and throws without verbal prediction. A small performance difference between the two conditions combined with high prediction accuracy should indicate ease of conscious access, as the access does not affect throwing performance in this case. In addition, verbal prediction costs might also manifest themselves in longer verbal response latencies and lower response volumes due to hesitation and uncertainty, a relation that has been observed in a word/shape classification task [44],in questionnaire response latencies [45], and in responses to trivia questions [46].

## 2 Materials and methods

### 2.1 Participants

Thirty participants (18 female, 12 male) from the student population of the Justus Liebig University, Giessen, Germany with an average age of 24.13 (*SD* = 5.77) years participated in the study. Participants were healthy and had normal or corrected-to-normal vision. Two left-handed subjects practiced the task with the right hand, which had been shown to produce similar learning curves to right-handed participants in pilot studies. Participants received course credit or monetary compensation of €8 per hour. The experiment was conducted in accordance with the ethical standards laid down in the Declaration of Helsinki. The protocol was approved by the Ethical Review Board of the Justus Liebig University, Giessen, and subjects gave written informed consent to participate in the study.

### 2.2 Experimental task and apparatus

**Fig 1:**
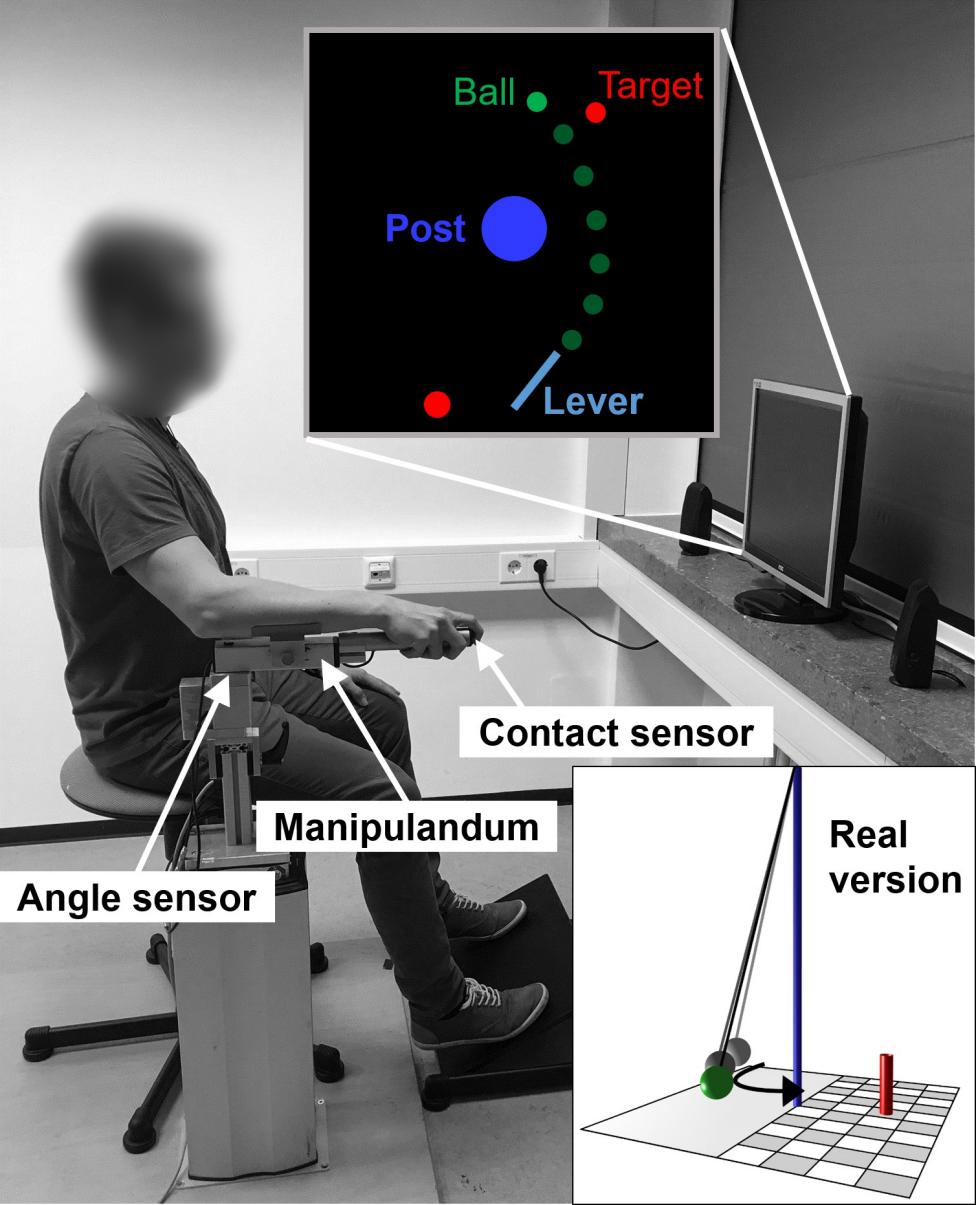
Experimental setup of the Skittles task. The participant uses a manipulandum to throw the virtual ball (green) with a horizontal rotational movement. The ball is released by lifting off the index finger from a contact sensor at the tip of the manipulandum. The ball travels on an elliptical pathway around the blue post to hit a red target. The individual on the picture has given written informed consent to publish these case details.

Participants practiced a novel and complex goal-oriented throwing task that has previously been used to study motor learning (e.g., [22,47–49]. The task is inspired by the British pub game “Skittles”, where a ball attached to the top of a post by a string has to be swung around the post to hit target objects on the opposite side. In addition to the ballistic nature of the task preventing online corrections during movement execution, this throwing task allows a temporal separation of movement execution and its terminal outcome, because the outcome is temporally delayed with respect to the movement. The task was executed semi-virtually. That is, participants executed a real ballistic throwing movement using a metal lever device (manipulandum), while the movement and its outcome were only visible on a computer screen from an overhead perspective (see Fig 1).

The experimental software was created using MATLAB R2018a (The Mathworks, Inc.) and the Psychophysics Toolbox version 3.0.14 [50]. On the screen in front of each subject, a virtual equivalent of the metal lever was displayed, which participants used to pick up and throw a green virtual ball (radius on screen = 4.2 mm) around a blue center post (radius on screen = 21 mm) in order to hit a red target (radius on screen = 4.2 mm). The elliptical trajectory of the ball around the center post was defined by the angle and velocity of the manipulandum at the moment of ball release. The calculation of the ball trajectory was based on a physical model of the task [48] with the following parameters: center post (radius = 0.25 m; position: x = 0.0 m, y = 0.0 m), target (radius = 0.05 m; position: x = 0.8 m, y = 0.9 m), ball (radius = 0.05 m; mass = 0.1 kg), spring constant (1.0 N/m). In the regular version of the task, participants were able to see the ball moving towards the target after ball release. Whenever the minimum distance between the trajectory of the ball center and the center of the target (D_min_) was less than or equal to twice the radius of the ball/target, the ball collided with the target (hit). For the subjects, this was apparent visually, because the target was pushed away from its position, and acoustically by the sound of two colliding billiard balls. In trials where D_min_ was larger than twice the radius of the ball/target, the ball missed the target. In the experimental version, in every second trial the ball vanished from the screen immediately after its release from the virtual lever. In these trials, subjects did not receive any information about the movement outcome.

### 2.3 Study procedure

Task execution was accomplished as follows: Participants sat on a stool placed 100 cm in front of a 19-inch, 4:3 computer monitor (model: Dell P190St, screen resolution: 1280 x 1024 pixels, refresh rate: 60 Hz). Their right arms rested on the foam padded manipulandum, which was fixed on a height-adjustable stand at the vertical rotation axis below the elbow joint of the participant (Fig 1). An integrated magnetic angle sensor with a resolution of 12 bit (0.09 deg) measured the lever rotation with a sampling rate of 1000 Hz. Movement was restricted to the horizontal plane, more specifically, to rotation around a fixed vertical axis. To pick-up the virtual ball, participants placed their index fingers on an electrical contact sensor at the tip of the lever to close an electrical circuit. They then “threw” the ball by moving the manipulandum in an outward horizontal movement similar to a Frisbee toss, and starting in front of their bodies. As soon as the participant’s finger was lifted from the manipulandum, the virtual ball was released from the virtual lever. To explain the task to the participants, a miniature model of the real Skittles game was used to clarify the task. To prevent a fast, rhythmic execution of subsequent trials, participants were instructed to start every trial by moving the tip of the virtual lever into a red circle positioned left of the fixed end of the lever (35° clockwise relative to the horizontal axis; see Fig 1). Immediately after the tip of the virtual lever reached the circle, it turned yellow. The circle turned green when the lever was held steady within the yellow circle for one second. The green starting circle signaled that participants were free to start the movement at any time. Note, however, that the subjects did not start the movement in reaction to the green signal. The aim of the task was to hit the target as frequently as possible.

The movement result was to be predicted verbally by the subjects within 2.5 seconds after ball release by the German words for hit (“Treffer”) or miss (“Fehler”). The verbal utterances were recorded for later analysis. For this purpose, a clip-on microphone (Monacor ECM-501L/SK), a phantom power adapter (MG STAGELINE EMA-1), and a microphone preamplifier (IMG STAGELINE MPA-202) were used. The output signal from the preamplifier was captured using a 16-bit data acquisition device (National Instruments PCIe-6321), and Matlab Data Acquisition Toolbox time synchronized with the data from the Skittles apparatus (angular and touch sensor) with a sampling frequency of 10.000 Hz.

### 2.4 Study design

The experiment took place over two sessions with 500 trials each. Trials were categorized with respect to practice and experimental conditions (overview in Table 1). The first 100 trials of session one served as a first practice of the task. The regular version of the task was used for all of these trials. From trial 101 to trial 150, the experimental version was used where ball flight information was masked for every second trial as described above (“Experimental task and apparatus”). In these trials, participants did not receive any outcome feedback, while feedback was normally presented in the other 50% of trials. This was done because performers usually drift away from a currently successful task strategy when deprived of visual feedback [51]. As this was also observed in pilot data, it was decided to provide visual feedback on every other trial. Thus, from trial 151 to trial 1000, the regular version of the task was alternated with the experimental version in every other trial and, additionally, participants were asked to verbally predict the outcomes of their throws in the trials without ball flight information and outcome feedback (*prediction condition*). Trials 151–200 served as practice of the verbal prediction. Only trials 201 to 1000 were used for analyses where the *prediction condition* (verbal prediction and no outcome feedback) was contrasted with the *regular condition* (no verbal prediction, but available outcome feedback).

**Table 1:** Overview of the experimental procedure.

	Trials 1–100	Trials 101–150	Trials 151–200	Trials 201–1000
**Experimental phases**	Practice: Regular task version	Practice: Alternation of regular task version and experimental version in every trial	Practice: Alternation of regular task version and prediction condition in every trial	Experiment: Alternation of regular task version and prediction condition in every trial
**Implementation of feedback and verbal prediction in the different phases**	100% of trials with outcome feedback	50% of trials with outcome feedback and 50% of trials without outcome feedback	50% of trials with outcome feedback and 50% of trials without outcome feedback and with verbal prediction	50% of trials with outcome feedback and 50% of trials without outcome feedback and with verbal prediction

As already mentioned, participants were not natural experts in the experimental task. But, previous studies using a similar version of the task showed that constant high hit rates are possible after 200 trials of practice and that hit rates vary between participants (e.g., [12]. It can therefore be assumed that relatively high levels of performances are achieved in the task. Yet, despite equal practice time for all participants, considerable variance in performance across participants is expected, based on varying personal state variables contributing to performance (like motivation, prior motor experiences etc.). Thus, variances in performances in the present study were taken as indicative of different skill-levels.

### 2.5 Analysis of throwing performance

Behavioral analyses as well as the analyses of verbal responses were done in MATLAB R2020b (The Mathworks, Inc.). The execution variables (release angle and velocity) and outcomes in the Skittles task are related in a nonlinear fashion. Furthermore, the task is redundant, what means that hits and misses are not functions of a dichotomous difference in throwing execution, but can arise from very different combinations in release angle and velocity. As a consequence, outcome prediction is not trivial. To account for this difficulty, only clear target hits (with D_min_ ≤ 7 cm) and clear misses (with D_min_ ≥ 12 cm) were analyzed (following [12]. Throwing performance and thereby skill-level was defined as the rate of clear hits in percent of blocks of 100 trials (10 blocks in total) averaged over all participants. Since the verbal predictions could influence throwing performance, hit rates between the *prediction condition* and the *regular condition* were compared. In these cases, hit rate was determined over 50 trials of each block for both conditions, because prediction trials and regular trials were alternated every trial.

### 2.6 Analysis of verbal responses

Verbal predictions of throwing outcomes were examined along two dimensions: First, prediction accuracy was analyzed in order to test whether participants were able to consciously access their internal processing of movement errors. Second, characteristics of speech, concretely variance in the onset and amplitude of verbal responses provided further information about the ease of conscious access to the predictions. As mentioned in the Introduction, it was assumed that faster (easier) conscious access would be manifested in earlier and louder prediction responses [44–46].

#### 2.6.1 Prediction accuracy

Prediction accuracy was defined as the rate of correct predictions relative to an individual baseline or chance level. This was accomplished in several steps. First, the rate of correct predictions (%C_Pred_) was defined as the percentage of correctly predicted clear hits and misses of all trials in the *prediction condition*. This empirical prediction rate was compared to the individually calculated prediction baselines (%C_Chance_). This baseline depends on two factors: first, the rate at which participants actually hit (Act_Hit_) or missed (Act_Miss_) the target in the experimental condition and, second, the rate at which they verbally report hits (Verb_Hit_) or misses (Verb_Miss_).

Based on the null hypothesis that verbal estimates are unrelated to the actual occurrence of hits and misses, we estimated %C_Chance_ in the following way:

%C_Chance_ = (Act_Hit_ * Verb_Hit_ + Act_Miss_ * Verb_Miss_) * 100

Prediction accuracy (%Acc_Pred_) was then computed as the percentage of correct predictions above %C_Chance_, normalized with respect to perfect predictions:

%Acc_Pred_ = (%C_Pred_ - %C_Chance_)/(100 - %C_Chance_)

Thus, %Acc_Pred_ represented the ability of participants to verbally predict the outcomes of their throwing movements.

#### 2.6.2 Speech characteristics

To analyze verbal utterances, voltage values from the microphone output were offset-corrected, rectified, and then a moving average calculation (window width 250 values, i.e. 25 ms) was performed. The onset time of a verbal utterance was identified when the averaged profile exceeded 0.1 V. To determine the amplitudes, the maximum value in the averaged voltage curve was first determined. To account for general differences in loudness resulting from different positioning of the microphone and speaking volumes of subjects, all maximum amplitude values of each test session and subject were divided by their medians. Finally, the medians of onset latencies and response amplitudes were determined for all trials in the four different categories Act_Hit_/Verb_Hit_, Act_Hit_/Verb_Miss_, Act_Miss_/Verb_Hit_, Act_Miss_/Verb_Miss_.

### 2.7 Statistical analyses

Statistical analysis was performed in JASP (Version 0.14.1). The alpha level was set to .05 for all statistical analyses. Data normality was checked with the Shapiro-Wilk test, and sphericity was checked using the Mauchly’s W test. In case of violation of sphericity, the Greenhouse-Geisser correction was used. Changes in performance were tested with repeated measurement ANOVAs over all sessions, which included *Holm* corrected post-hoc testing of single sessions. Throwing performance in the two experimental conditions (*regular* vs. *prediction*) was compared using the Wilcoxon signed-rank test (due to violation of normality assumptions) and the rank-biserial correlation coefficient as effect size to test for negative effects of verbal prediction (“verbal prediction costs”). A one-sample *t*-test was used to examine whether prediction accuracy (%Acc_Pred_) was above baseline prediction, with effect sizes determined by Cohen’s *d*. It was expected that prediction accuracy would be a function of forward-model quality and quality of conscious access. Hence, a Spearman correlation between prediction accuracy and hit rate as well as between prediction accuracy and the differences in hit rates between the *regular condition* and the *prediction condition* (verbal prediction costs) was conducted. Speech characteristics (response latency and amplitude) were tested by a 2 (actual hit or miss) × 2 (verbalized hit or miss) repeated measures ANOVA, with verbal prediction costs as a covariate. In addition, a Bayesian inference approach was used, with Bayes factors (*BF*) interpreted as the amount of evidence for the null and the alternative-hypothesis before versus after inspection of the data [52]. The size of the *BFs* were interpreted according to Raftery [53]. *BFs* 1–3 were interpreted as weak evidence for the alternative hypothesis against the null hypothesis, 3–20 as positive, 20–150 as strong, and *BFs* > 150 as very strong evidence.

## 3 Results

### 3.1 Throwing performance

**Fig 2:**
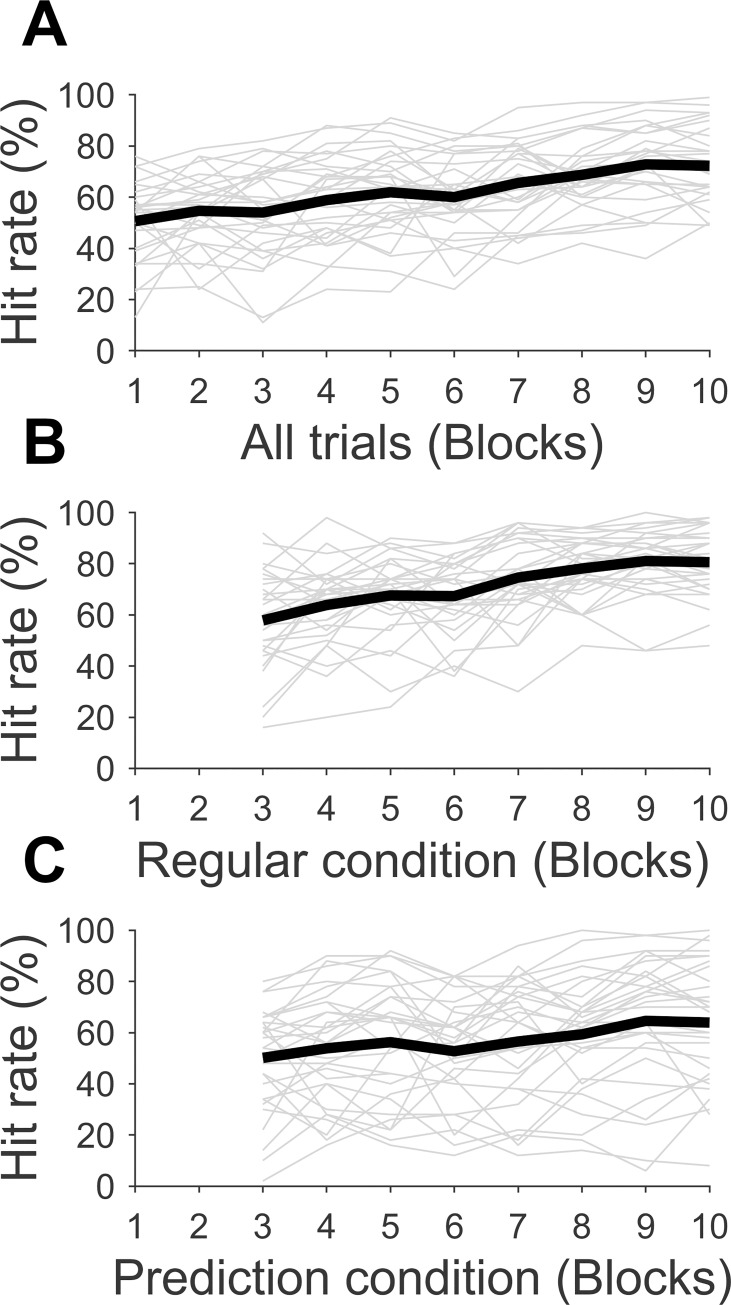
Development of throwing performance over all trials (A), over trials under the regular condition (B), and over trials under the prediction condition (C). Thick black lines represent the group average; thin grey lines represent individual data.

Fig 2 depicts the development of throwing performance (hit rate). Fig 2A shows the hit rate over all trials executed. In Fig 2B, only trials carried out under regular conditions are shown (from trial 201 on), and Fig 2C illustrates the hit rates of only those trials of the *prediction condition* (from trial 201 on). Hit rates started at around 50% on average, and rose with practice until they levelled off around block seven. ANOVA analyses carried out with repeated measures showed a significant main effect of block (*F*(4.65, 134.85) = 18.92, *p* < .001, *ɳ*_*p*_^*2*^ = .40, *BF*_*10*_ > 150). Post hoc tests revealed significant differences between blocks 1–6 and block 10, practically no differences between block 7 and 10, and no differences between blocks 8, 9 and 10 (see Table 2). Interindividual variance was relatively large in general (standard deviation of the hit rate of all trials was 13.49%, see Table 3), and even larger in the *prediction condition* (SD = 22.71%). The average hit rate also differed significantly between the *regular condition* and the *prediction condition* (*W* = 61, *p* < .001, *rank-biserial correlation* = -.74, *BF*_*10*_ > 150), but there was also large variance between participants (SD = 20.73%, Min = -5.24%, Max = 60.33%).

**Table 2:** Post-hoc comparisons between blocks 1–9 and block 10.

Session	Hit rate Mean difference	SE	t	p_holm_	BF_10_
1 vs. 10	-0.22	0.03	-8.58	< .001	> 150
2 vs. 10	-0.18	0.03	-6.98	< .001	> 150
3 vs. 10	-0.19	0.03	-7.24	< .001	> 150
4 vs. 10	-0.14	0.03	-5.31	< .001	> 150
5 vs. 10	-0.11	0.03	-4.10	< .001	> 150
6 vs. 10	-0.01	0.03	-4.85	< .001	> 150
7 vs. 10	-0.13	0.03	-2.66	.15	6.094
8 vs. 10	-0.07	0.03	-1.40	1.00	1.760
9 vs. 10	-0.04	0.03	0.23	1.00	.210

SE = Standard error, holm = Holm correction.

**Table 3:** Descriptive data of hit rates in the two experimental conditions and both conditions together (All trials).

	N	Mean	SD
Hit rate *All trials*	30	64.28	13.49
Hit rate *Regular*	30	72.26	13.77
Hit rate *Prediction*	30	56.00	22.71
Difference between *Prediction* and *Regular*	30	16.27	20.73

SD = Standard deviation.

### 3.2 Prediction accuracy

**Fig 3:**
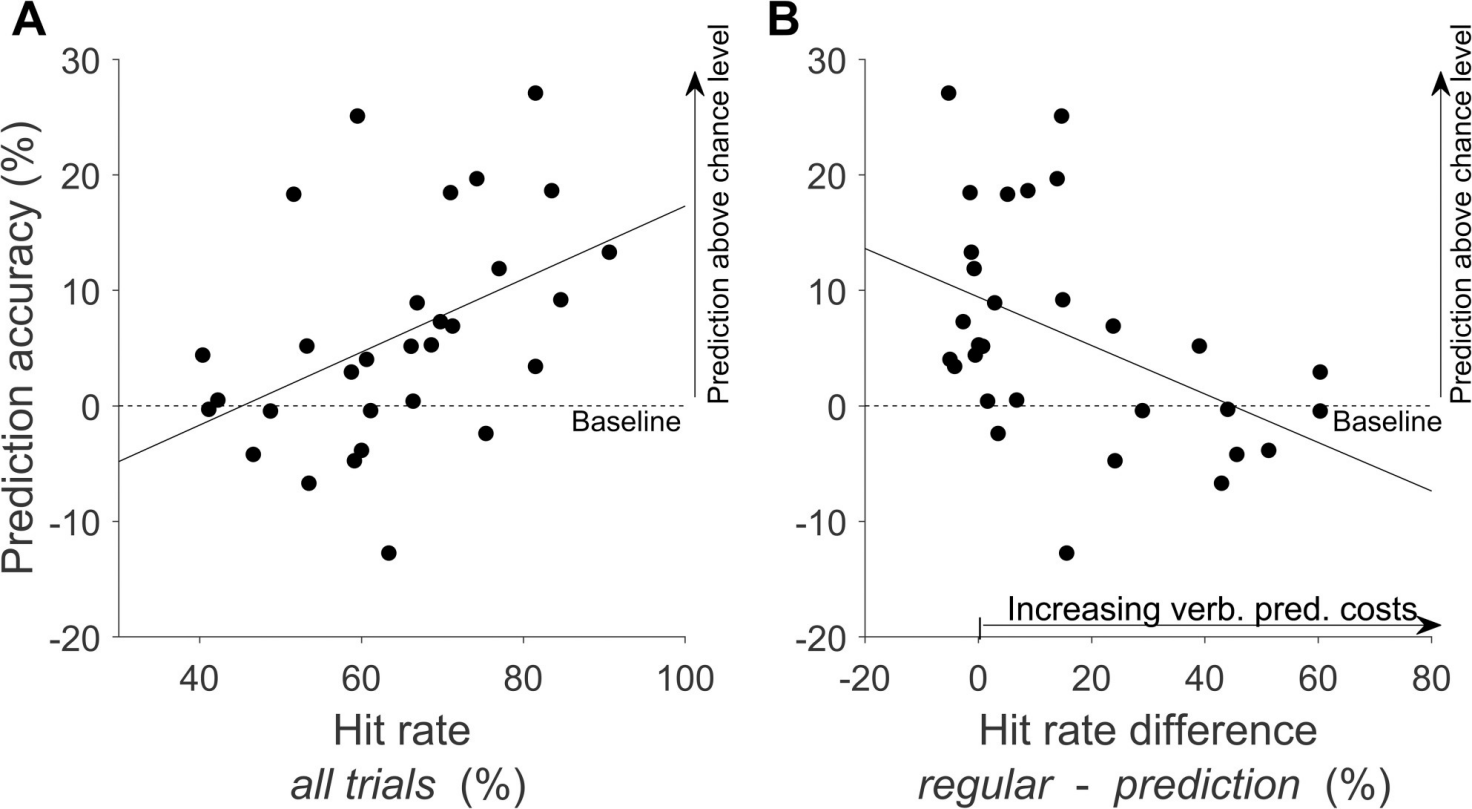
Correlations of throwing performance with prediction accuracy relative to individual chance level (see 2.6 “Analysis of verbal responses”). Each dot represents the average values of a single participant relative to individual chance level (represented by the dashed line).(A) Correlation of prediction accuracy with hit rates over all experimental trials (*prediction condition* and *regular condition* together). (B) Correlation of prediction accuracy with the difference in hit rates between the *prediction condition* and the *regular condition* (verbal prediction costs). The larger the difference value, the lower the performance in the *prediction condition* (and the higher the prediction costs).

The prediction baseline was on average 55.41% (SD = 12.83%), which corresponds to the average chance level for the participants’ predictions. Average prediction accuracy (%ACC_Pred_) exceeded the prediction baseline by 6.01% (SD = 9.61%) of the potential accuracy gain by predicting, which was significant (*t*(29) = 3.42, *p* = .002, *d* = .63, *BF*_*10*_ = 19.19). Prediction accuracy, however, varied greatly between participants (see Fig 3). There was a significant positive correlation of %Acc_Pred_ and hit rate over all trials (*r* = .44, *p* = .014, *BF*_*10*_ = 4.03; Fig 3A). Since throwing performance differed between the *regular condition* and the *prediction condition* with large variance between participants, it was tested whether this variance also accounted for the differences in prediction accuracies. A significant negative correlation of %Acc_Pred_ with the difference in hit rate between the *regular condition* and the *prediction condition* was found (*r* = -.52, *p* = .003, *BF*_*10*_ = 4.62; see Fig 3B). This means that participants with lower hit rates in the *prediction condition* relative to the *regular condition*, and thus higher verbal prediction cost, showed poorer prediction accuracy, in the lowest cases even below baseline level.

### 3.3 Speech characteristics

**Fig 4:**
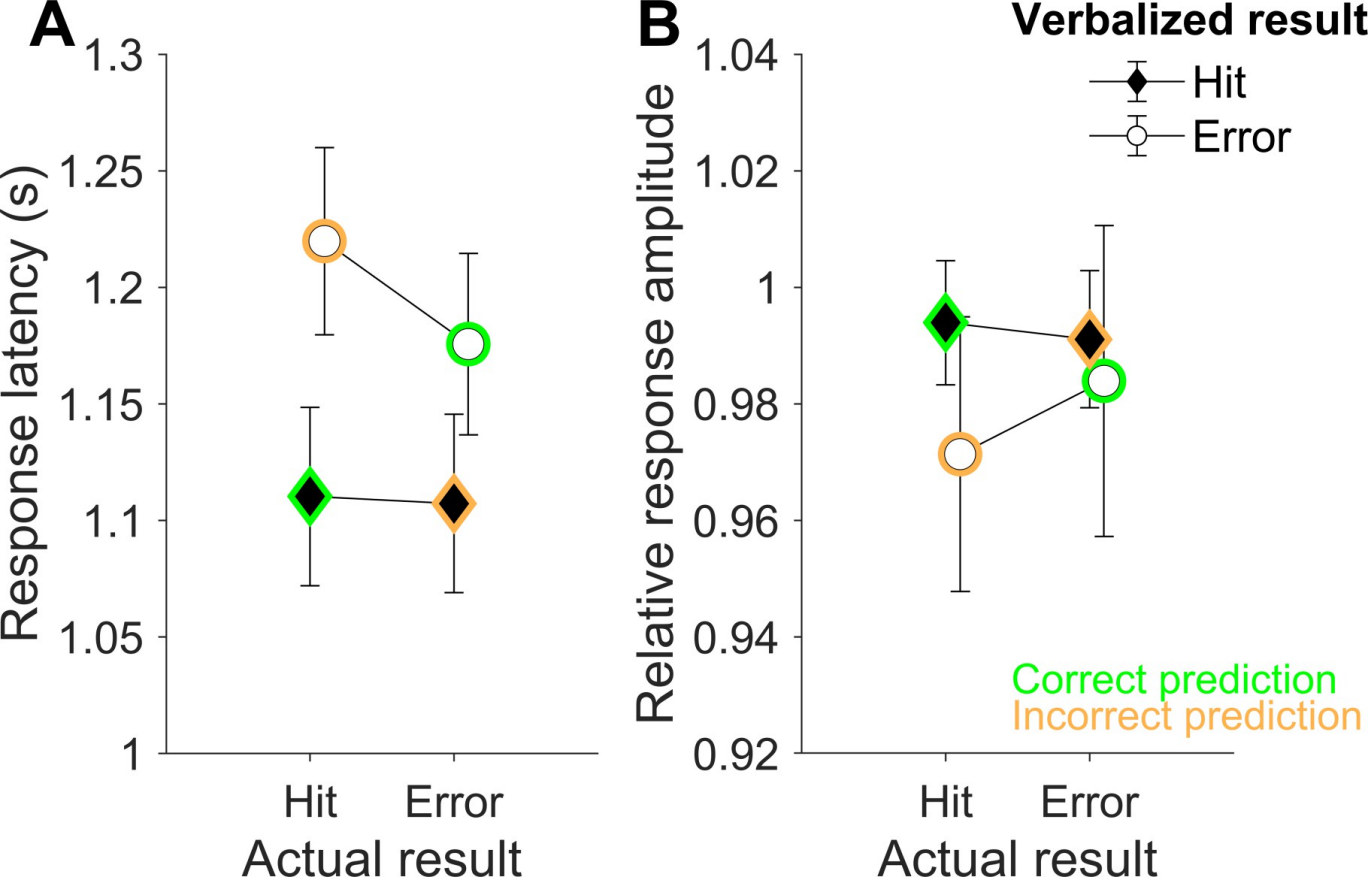
Speech characteristics of verbalized predictions. Average response latencies (A) and response amplitudes (B) differentiated after actual results of trials (actual hits or misses) and the verbalized responses (verbalized hits or misses). Error bars represent standard errors of the mean. Correction predictions are marked in green, incorrect predictions are marked in orange.

Fig 4 shows the response latencies (A) and amplitudes (B) with respect to the *verbalized prediction as a function of actual outcome*. It can be observed that responses predicting misses were generally slower, and tended to also be quieter, than responses predicting hits. The latency difference was confirmed by a main effect regarding the *verbalized result* (*F*(1, 28) = 12.90, *p* = .001, *ɳ*_*p*_^*2*^ = .32, *BF*_*10*_ > 150), but there was no main effect of *verbalized result* for the amplitude variable (*F*(1, 28) = 0.79, *p* = .38, *ɳ*_*p*_^*2*^ = .03, *BF*_*10*_ *=* .26). There was also a small main effect for *actual result* in response latencies (*F*(1, 28) = 4.78, *p* = .037, *ɳ*_*p*_^*2*^ = .15), which could, however, be ascribed to an interaction effect (see below). Bayesian statistics confirmed that the data was not sufficiently informative to allow a strong conclusion to be drawn about the main effect *actual result* regarding the latency variable (*BF*_*10*_ = .434). Nevertheless, verbal predictions of trials that were incorrectly predicted as misses were clearly voiced later than predictions of trials correctly predicted as misses, and this difference was not observable in the trials predicted as hits. Classical ANOVA revealed an interaction effect between *actual result* and *verbalized result* (*F*(1, 28) = 5.41, *p* = .028, *ɳ*_*p*_^*2*^ = .16). A Bayesian mixed-factor ANOVA also determined that the data were well represented by a model that included both main factors, *actual result* and *verbalized result*, and the *actual* × *verbalized* interaction. The *BF*_*10*_ was 7459, indicating decisive evidence in favor of this model when compared to the null model. Moreover, the *BF*_*10*_ in favor of indicating the interaction effect on top of the main effect *actual result* was 5.82. A similar interaction (incorrectly predicted misses seem to be expressed most quietly) could neither be confirmed by classical nor by Bayesian ANOVA (*F*(1, 28) = .57, *p* = .46, *ɳ*_*p*_^*2*^ = .02, *BF* = .01). There were also no interactions with the covariate *performance loss* (differences in hit rates between the *regular condition* and the *prediction condition*) in the amplitude results.

**Fig 5:**
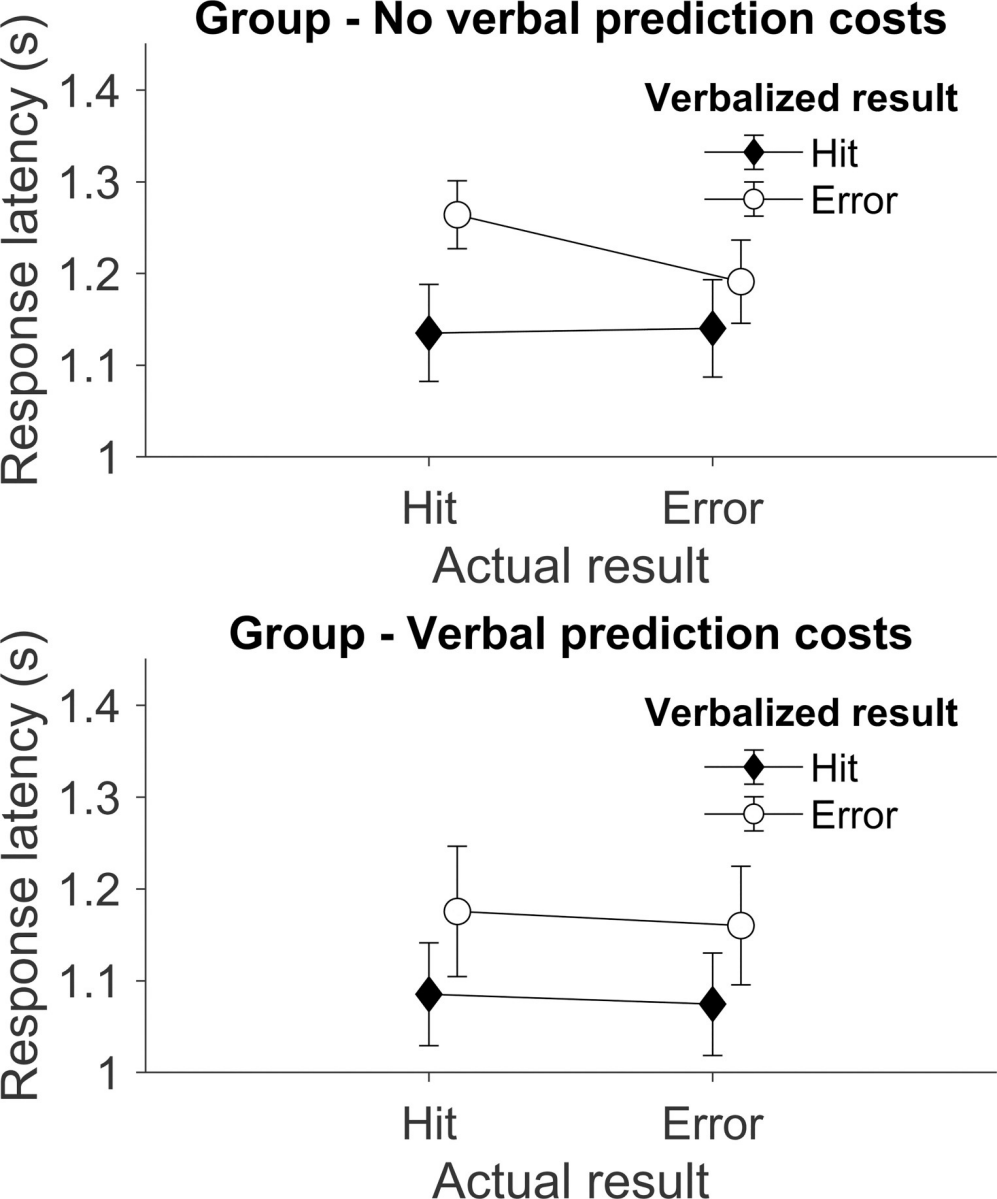
Different response latency effects depending on verbal prediction costs. Average response latencies differentiated after actual results of trials and verbalized results for two groups separated based on whether they experienced verbal prediction costs (bottom) or not (top). Error bars represent standard errors of the mean.

Regarding response latency, the three-way interaction with the covariate *actual result* × *prediction* × *verbal prediction costs* did not achieve significance, but showed a trend for a different interaction depending on the size of performance loss in the prediction condition, i.e. the level of verbal prediction costs (*F*(1, 28) = 5.50, *p* = .07, *ɳ*_*p*_^*2*^ = .11). In addition, the Bayes model, including the main factors *actual result* and *verbalized result* and the *verbal prediction costs* covariate, strongly outperformed the null model (*BF*_*10*_ > 150), and weakly outperformed the model including the *actual* × *verbalized* interaction (*BF*_*10*_ = 1.48). To analyze how this trend was supported by the data, a median split was applied with respect to *verbal prediction costs*: one group showed virtually no verbal prediction costs (< 7.76%) and the other experienced verbal prediction costs (> 7.76%). Fig 5 illustrates that the interaction between *actual result* and *prediction* exists only in the group of participants who showed no verbal prediction costs.

## 4 Discussion

The present study examined whether subjects with relatively high skill in a motor task can verbally predict outcomes of their own actions prior to receiving any external feedback about action outcomes. To this end, participants practiced a virtual goal-oriented throwing task for 200 trials. In every other of the remaining trials, they were then asked to verbally predict whether the ball they had just released would hit or miss the target (*prediction condition*). They had 2.5 seconds after releasing the ball to make their predictions. Vision was occluded immediately upon ball release and no feedback was given about the trajectory of the ball. In the other half of the trials, participants did not have to predict outcomes and they could see the ball moving towards the target and hitting or missing it (*regular condition*). Results in terms of throwing performance, prediction accuracy, and speech characteristics of verbal responses were analyzed and compared between the *prediction condition* and the *regular condition*.

### Conscious access to outcome predictions is possible, but varies interindividually

On average, prediction accuracy (as the measure of conscious access to outcome predictions) exceeded baseline levels by about 6% of the potential gain in accuracy. This means that 6% of what, in theory, could have been achieved above chance when using internal information was achieved. 100% in that measure would indicate predictions where every trial is correctly classified as error or hit, while 0% represented the prediction accuracy that can be achieved when using an optimal base rate, but without any additional information provided by a conscious access to internal prediction processes. Without such additional information, verbalized reports of anticipated outcome predictions could only be made at the chance level. Chance level was reflected in the individual baseline level, taking into account actual hit rates (Act_Hit_ and Act_Miss_) and verbal report rates (Verb_Hit_ and Verb_Miss_). Since this is the optimum that can be reached by simply matching the ratio of verbal responses with an estimate of one’s individual hit rate, prediction accuracies above this level necessarily require the use of information from an individual throw. Any improvement above baseline level must have been driven by information gathered during the throwing movement. As no external visual information about the ball trajectory was available, this additional information needed to be derived from internal sources including correlates of efferent commands.

Variance of above-baseline gains in accuracy prediction was relatively large between participants, with some individuals achieving gains of around and above 20%, while others fluctuated around chance level. As described in the introduction, predictive accuracy is assumed to be a function of prediction quality (quality of a task specific forward model) and ease or efficiency of conscious access to the forward model predictions. So, individuals with higher prediction accuracies should have relied on both, a sufficiently good prediction quality as well as an easy and accurate access to their internal processes. Conversely, the reason for poorer prediction accuracy may have been poorer prediction quality and/or more difficult access to internal processes. Thus, verbal predictions of outcomes of complex motor actions, such as basketball shots, are possible but vary widely. Our data do not allow a clear separation between prediction quality and ease of access to explain this variance. But, we will discuss the possibility to disentangle the two factors based on our behavioral and vocalization results (especially comparing the *regular* and the *prediction* condition) and embed this into existing knowledge about motor skill and prediction skills.

### Prediction accuracy depends on skill level and verbal prediction costs

Quality of prediction has already been associated with experience or skill. Forward model computations contribute to learning [19], and extended experience in a motor task correlates with distinct signs of predictive error processing on the neurophysiological level [12,13,20–22]. Furthermore, it has been shown that motor experts (sports athletes) can anticipate the outcomes of other players’ actions with relatively little information about action parameters, and that this ability rises with skill level [24–26,30]. In these studies, temporal occlusion paradigms were used, where participants with varying expertise levels watched videos of motor actions and had to predict the outcomes of the actions shown at different points in time. In the Aglioti and colleagues’ study [24], professional basketball players were capable of correctly predicting shooting outcomes above the level of chance even before players on the videos released the ball. These motor experts were contrasted with participants with high visual experience (sports journalists and basketball coaches). Pure visual experts needed significantly more information to correctly judge the outcomes above the level of chance. This difference indicates that motor expertise facilitates perceptual abilities in general and, more specifically, at least partially through predictive functions. The studies of Maglott et al.35 [35], Cañal-Bruland and colleagues 34 [34], and Gray et al.36 [36] extended these findings to predictive abilities with respect to subjects’ own movement outcomes, but with some methodological issues which we tried to resolve in the present study by considering individual success rates, response strategies, and the information used for predictions. Although experience and expertise in the task used here was not comparably high to natural experts in terms of practice trials, participants reached a learning plateau, and similar studies with the same task have shown that hit rates of more than 60% coincided with a neurophysiological marker of forward model predictions [12,13]. In the present study, participants reached an average hit rate of over 50% within the first 100 trials, and increased their hit rate average to 72% in the last 100 trials, taking into account both experimental conditions (*prediction* and *regular*). In the *regular condition* alone, they even reached a hit rate of over 80%. But, interindividual variance in performance was relatively high. Since it is suggested that prediction quality of forward models and learning/performance are interdependent [19,22], it can be assumed that individuals with higher hit rates have a better forward model of the task and thus a better prediction quality than individuals with lower hit rates. And this is indeed reflected in a positive correlation between prediction accuracy and hit rates in the present study.

The second factor contributing to prediction accuracy, ease of access, can be captured when comparing performances in the *regular condition* with the *prediction condition*. There was a clear difference in hit rates between the *regular condition* and the *prediction condition*. Hit rates in the *regular condition* were on average higher, but there were again large differences between participants. While about half of the participants did not show much change in hit rates between the two conditions, the other half experienced large decreases in performance when throwing outcomes had to be predicted verbally (i.e., they had a high verbal prediction costs). This difference in hit rates between the *regular condition* and the *prediction condition* correlated negatively with prediction accuracy. Prediction accuracy was higher when verbal prediction costs in the *prediction condition* were lower. This means that the experimental demands of the *prediction condition* affected motor task performance more in some individuals than in others. What may be the reason for this? During the experimental procedure, participants had to respond verbally no later than 2.5 seconds after releasing the ball. Hence, preparation of the response and the focus on accessing relevant internal information for the outcome prediction might have interfered with motor control processes, leading to a performance loss in the *prediction condition*. This observation is in line with well-established findings that have shown that attention to performance can become counterproductive [42,43,54,55]. Thus, the less-skilled participants in the present study were more impaired by the verbal prediction requirement presumably because they had less effective access to information relevant for outcome predictions, and needed to direct their attention to motor execution, which interfered with their performance. The results from analysis of the speech characteristics of the verbal responses further support this interpretation.

### Response latency is related to throwing performance and prediction accuracy

Speech production is sensitive, and hesitation and uncertainty of responses can be observed in measures of response latency and amplitude [44–46]. Hence, possible detrimental effects of verbal predictions of movement outcomes [41] may be represented by these variables. Movement outcome predictions are based on different sources of information gathered during movement planning (efference copy) and movement execution (haptic, proprioceptive, or visual information; [10]), with each of these modalities producing different time delays and resulting in varying degrees of accuracy [56–58]. It can be assumed that outcome estimates are continuously produced, resulting in an increase in accuracy of the input information while the movement is evolving. Hence, responses can be quick if sufficiently accurate information is available early on, or if predictions are, instead, based on experience, or are “thoughtlessly” uttered without the subject’s consideration of the actual input information when making predictions.

In the present study, there was a general observation of quicker responses when a hit was predicted, irrespective of whether this prediction turned out to be true or not. In contrast, response latency differed between correctly and incorrectly predicted misses: Predictions of trials that were incorrectly predicted as misses were verbalized more slowly than predictions of trials correctly verbalized as misses. This difference was only observable in participants without verbal prediction costs in the *prediction condition*. Moreover, correctly predicted hits and correctly predicted misses had more similar response latencies (and amplitudes) than incorrectly predicted trials, especially in participants without verbal prediction costs. Since outcome predictions are generated based on available efferent information about motor commands and incoming sensory information about movement execution, this latency effect in relation with the group differences may provide indications regarding the ease of access of internal predictions processes. In cases of correct predictions, input information may have been clear and accurate relatively early, leading to relatively fast outcome estimates. In contrast, incorrectly predicted misses resulted in slower response latencies. This indicates that the information gathered here was more ambiguous for outcome predictions, and that this ambiguity did not resolve over time. As a result, participants hesitated, waited longer, apparently still hoping for better information resolution, and ended up answering incorrectly. This effect was not observed in participants with performance decrements when verbally predicting. They had similar response times between correctly and incorrectly predicted trials. Hence, they showed no signs of hesitation (slow response) or, on the contrary, confidence (fast response). This suggests that these participants were apparently unable to differentiate between accurate and ambiguous efferent/afferent input information available to the motor system for prediction, which may be explained by a limited access to internal prediction processes.

As already described, the latency effect was generally not present in incorrectly predicted hit trials. The reason for the difference between hit and miss predictions could be a general bias toward hit responses, as has been observed in other studies [34,35]. Response bias in the computation of prediction accuracy was controlled for, but it may have still been inherent in verbal responses. Hence, it is possible that responses predicting hits were expressed based on experiences instead of waiting for accurate input information, which led participants to respond (too) early. However, further experiments would be needed to confirm this assumption.

Taking together results of prediction accuracy, throwing performance, and speech characteristics of verbal responses, it can be concluded that a higher level of task skill allows faster access to accurate motor predictions without interference with motor control processes. On the contrary, when throwing performance is poor, conscious access to internal predictions negatively affects movement execution, presumably in the form of verbal prediction costs. In light of these results, the question posed in the title can be answered in the affirmative: Yes, he can! Given the fact that Stephen Curry has exceptionally good throwing skills, it can be assumed that he has an excellent forward model and good access to internal predictions.

### Limitations

There are limitations in this study that need to be taken into consideration when interpreting results. First, as already mentioned, participants in the present study are not comparable to natural motor experts like NBA players. Nevertheless, especially those participants with quite high performances were able to verbally predict their own throwing outcomes above chance level. Thus, differences in prediction accuracy could be explained by differences in skill level. Second, no neurophysiological measures were recorded, which could have provided more direct information about internal prediction processes. Temporal characteristics of error-related potentials in electroencephalogram measures, such as the error-related negativity [6,7] could have supported interpretations about the response latency effects. Third, there might be another explanation for the variances in prediction accuracy aside from verbal prediction costs that cannot be fully ruled out: In the prediction condition, participants had no visual feedback about throwing effects (i.e., vision was occluded as soon as the ball was released). Although feedback in the form of information about ball trajectory could not have had any influence on performance in the current trial (since the throwing movement was already terminated at that point), such feedback might have affected the participants anticipatively. That is, knowing that a trial would be without feedback could have unsettled and blocked them. This explanation is regarded as not very likely, but only an experimental separation of missing visual feedback information about action outcomes from verbal prediction costs can provide a clear differentiation.
